# Implementing cheminformatics

**DOI:** 10.1186/s13321-019-0333-z

**Published:** 2019-02-05

**Authors:** Rajarshi Guha

**Affiliations:** Vertex Pharmaceuticals, 50 Northern Ave, Boston, MA 02210 USA

## Content

Computational characterization of chemical structures originated before the advent of digital computers [1]. However, the ability to represent and manipulate large collections of molecules and their associated information was enabled by the rise of cheminformatics algorithms and their implementions on digital computers. Willett [2] has suggested the work of Ray and Kirsch [3] on substructure searching as the first description of a computer implementation (on punched cards) of a cheminformatics algorithm.

Programming language research blossomed during the 1950’s and 60’s and saw the development of high level programming languages (such as FORTRAN [4], LISP [5] and ALGOL [6]). Cheminformatics research took advantage of these efforts, to move beyond punched cards. One of the earliest cheminformatics applications in a high level language was DENDRAL [7], written in LISP in 1963 [8]

Since the 1960’s, a plethora of languages have come into existence. Each language has its distinct features (directly memory manipulation in C, code as data in LISP [9], automated memory management in Java, lazy evaluation [10] in Haskell), but useful features from one language tend to show up in others (e.g., automated memory management initially appeared in LISP, but is now found in Java, Ruby, Python, C# and others). Furthermore, all modern languages are Turing equivalent [11] (i.e., capable of performing any arbitrary computation). One might then ask, what does it matter what language one uses to implement cheminformatics?

A number of factors go into deciding what language to use in a given setting. These include the suitability for a specific task (web development versus statistical modeling), prior knowledge of the language, the availability of supporting tools & frameworks and their licensing requirements and of course, performance.

A key consideration is the availability of external libraries such as cheminformatics toolkits (e.g., CDK [12] or JChem for Java applications). Many libraries (especially those written in C or C++) can be wrapped and made accessible to other languages (e.g., OpenBabel [13], RDKit and OEChem which are written in C++ provide SWIG wrappers enabling their use in Python and Java). Finally, for many projects, the choice of language is dictated by historical development (such as the use of Fortran for much of scientific computing).

At a more fundamental level, there are different programming models, which require conceptually different approaches to designing an application. For example, Khomtchouk et al. [14] suggest that the functional paradigm is best suited for scientific software development. On the other hand, Ray et al. [15] show that projects using functional languages do not necessarily show better software quality. One must consider others aspects, ranging from performance issues to the availability of programmers with sufficient skills to develop and then maintain applications written in functional languages. It is useful to note that some languages such as Scala are a hybrid, supporting both functional and procedural paradigms.

In this thematic series we have invited authors to present their views on a variety of programming languages. The series is rolling, and starts of with contributions from Thiesen [16], Berenger [17], and Höck [18] discussing JavaScript, OCaml and Scala respectively. We anticipate contributions covering Scala, C/C++, Tcl and noSQL.

The intended audience for this series are practitioners of cheminformatics who are already familiar with one programming language and would like to learn what other languages may offer in terms of language features and supported tooling.

We do not intend this to be a head to head comparison. Rather, the contributions are structured to address one or more of the following aspects

- How that language (or model of programming) affects scientific software development
- How a language may enable the development of new approaches to solving a problem in cheminformatics or computational chemistry
- Specific approaches to overcome language limitations when dealing with chemical of biological data types
- Comments on performance and it’s relevance to the languages goals
- Educational aspects of the language (is it easier for newcomers?)
- Development environments and frameworks that make a language easier to use and deploy (e.g., RStudio for R and Jupyter notebooks for Python)

The goal of this issue is to highlight features of different languages that the authors have employed to build applications as well as their views on the benefits (and downsides) of the language that has driven them to invest effort in building capabilities in their chosen language. We do not expect that this will identify any single language as the “chosen one”. Rather, we hope that the articles in this issue will be a useful guide for the community to assess which languages may be appropriate for their next project.
